# Mate-switching is not associated with offspring fitness in a socially monogamous bird

**DOI:** 10.1098/rspb.2025.0577

**Published:** 2025-05-28

**Authors:** Frigg Speelman, Terence Burke, Jan Komdeur, David Richardson, Hannah L. Dugdale

**Affiliations:** ^1^ School of Natural Sciences, Macquarie University Faculty of Science and Engineering, Macquarie Park, New South Wales, Australia; ^2^ Behavioural and Physiological Ecology, Groningen Institute for Evolutionary Life Sciences, Groningen, The Netherlands; ^3^ School of Biosciences, University of Sheffield, Sheffield, UK; ^4^ Biological Sciences, University of East Anglia, Norwich, Norfolk, UK; ^5^ Nature Seychelles, Mahé, Republic of Seychelles

**Keywords:** reproductive success, lifespan, telomere length, haematocrit, divorce, body mass, parental effects, pair bond

## Abstract

In many species, individuals form socially monogamous pair-bonds lasting multiple breeding seasons, or even whole lifetimes. Studies often suggest social monogamy to be adaptive, but this is usually quantified through the survival and annual reproductive success of the partners. However, beyond the number of offspring produced, parental partnerships may also affect their offspring’s phenotype, health and ultimately fitness. Using multigenerational data on the Seychelles warbler (*Acrocephalus sechellensis*), we investigated the impact of parental pair-bond tenure (pair-bond duration) and pair-bond ending (pair-bond ended across breeding seasons) on offspring fitness components. First, we addressed juvenile-stage fitness components using indicators reflecting physiological state (haematocrit, telomere length and body condition). Second, we assessed long-term fitness components using offspring lifespan and lifetime reproductive success (LRS). We found no consistent evidence of pair-bond tenure or pair-bond ending effects on short-term (telomere length, haematocrit and body condition) or long-term (lifespan and LRS) fitness components. To our knowledge, this is the first study quantifying long-term parental effects of pair-bond tenure and pair-bond ending on offspring fitness components in wild populations. This work provides insights into the lack of intergenerational implications of long-term socially monogamous partnerships.

## 1. Introduction

In many species, individuals form socially monogamous pair-bonds that are often maintained over multiple breeding seasons or even entire lifetimes (i.e. pair-bond fidelity). Especially in birds, social monogamy is common (approx. 80% of species) [1]. Sticking with the same partner over multiple breeding attempts can reduce sexual conflict, especially in long-lived species with biparental care where individuals have to cooperate to raise offspring [2,3]. Here, over-exertion of one partner in the current reproductive attempt will be detrimental for both partners if this reduces future reproductive investment. This means the evolutionary interests (i.e. in future reproductive success) of both individuals are more aligned within long-term than short-term partnerships. Additionally, pair-bond fidelity can reduce mate searching costs [4,5]. Finally, staying together for longer (i.e. increased pair-bond tenure) can improve coordination and familiarity between the pair [6,7]. This, in turn, can increase reproductive success, competitiveness and survival of pair-bonded individuals [7]. Many studies have found evidence of social monogamy being an adaptive strategy, but this is usually quantified by addressing the effects of social monogamy on the survival of the partners [8,9] and their annual reproductive success [10]. Additionally, studies on long-term social monogamy are lacking in cooperative breeders (but see [11], although they do not address offspring fitness effects). Especially in species with biparental care, partnership quality may ultimately affect the offspring’s phenotype and health. If newer partners are not yet well aligned or coordinated, offspring sired early in partnerships may suffer fitness costs (both in the short and long term) as a result of less than optimal parental care [2] and increased parental stress [12]. Partnership termination may also have detrimental effects on offspring condition and survival; especially when the partnership is terminated while offspring are still dependent or closely associated with their parents.

Partnership termination effects on offspring fitness can be defined as parental effects, whereby the parent’s phenotype causally influences their offspring’s phenotype beyond the genes they inherit [13]. These are often shaped by natural selection [14] and can both accelerate and decelerate evolutionary responses to selection since traits respond to both current (on the offspring) and previous (on the parents) selective forces [15]. In birds, direct parental effects arise through, for example, investment in the egg composition and production, provisioning and other forms of parental care [14]. Indirect parental effects can also occur if a parent themselves gains care from their mate (e.g. nuptial feeding) and also through the benefits of mate choice [14,16]. Parental effects can have a pronounced influence on offspring in early life when they are still dependent on their parents [17], and a growing number of studies have identified parental effects on offspring throughout their life (e.g. influencing offspring survival and lifetime reproductive success [18–21]). However, no studies to date have investigated how pair-bond ending and pair-bond tenure of the parents in early life affect offspring, with the exception of psychological and sociological studies on humans [22,23].

Here, we investigate the short- and long-term consequences of parental pair-bond tenure and pair-bond ending on offspring fitness using a 25 year dataset of a closed population of Seychelles warblers. First, we addressed juvenile-stage fitness using three indicators that reflect physiological state: offspring (i) telomere length, (ii) haematocrit and (iii) body condition. Second, we tested whether pair-bond ending was associated with offspring (iv) lifespan and (v) lifetime reproductive success (LRS). We hypothesize that parents in longer partnerships rear offspring in the better physiological state, which go on to have higher fitness. In addition, we expect that pair-bond disruption while the offspring are still dependent on parental care will negatively affect offspring physiological state and fitness. Offspring produced in the breeding season prior to pair-bond termination will probably experience physiological stress, as they are still dependent on parental care, and may be affected by potential stress expressed by the parent(s), as well as the disruption of group stability and potential subsequent social conflict in the breeding territory.

Seychelles warblers are long-lived (up to 21 years), are facultative cooperative breeders, and form socially monogamous partnerships in breeding territories that they defend year-round [24]. Partnerships can last from one breeding season up to at least 15 years, and can end through the death of a partner (widowhood), or less commonly divorce and human-induced translocation of one partner as part of a conservation programmes [25,26]. Both parents provide parental care through provisioning and nest guarding during a long period (approx. 3 months) of offspring dependence for an altricial bird species [27,28]. Parents can be accompanied by 1−5 sexually mature subordinates that may provide help through alloparental care when both parents are present [27,29], which increases provisioning rates at the nest [30]. The study system provides an excellent model system since it is an isolated population with virtually no migration [31] that has been extensively monitored over many generations. Therefore, we can accurately identify and track individuals of known age throughout their lives, generating extremely accurate survival estimates that are not confounded by dispersal [32,33]. Additionally, we have detailed information on territory residency and social status, thus, we can accurately identify partnerships. Individuals are captured and blood sampled throughout their lifetime; so we have access to individual measurements of physiological state. Finally, we have a genetically verified population pedigree [34], which allows us to accurately estimate LRS.

The juvenile-stage fitness proxies used in this study (telomere length, haematocrit and body condition) have already been shown to reflect the health and condition of Seychelles warblers. Telomeres—repetitive DNA sequences at the end of linear chromosomes—protect against DNA damage and can shorten due to oxidative stress [35]. In the Seychelles warbler, telomere length generally declines with age, especially in early life—although telomere lengthening also occurs [36]—predicts future mortality [37] and has been linked to genetic, parental and environmental conditions [34,36]. Importantly, in this species, telomere shortening indicates costs associated with inbreeding [38], social conflict [39], reduced parental care [29], food availability [40,41] and malaria [40]. Haematocrit, the proportion of blood comprised of erythrocytes, reflects aerobic capacity in vertebrates, which affects individual health and performance [42]. In the Seychelles warbler, high haematocrit indicates poor condition for juveniles [43]. Finally, body condition is often used as an indicator of physiological state in animals, as it is determined by body fat content (i.e. energy reserves) and structural size, and can affect survival [44,45]. Juvenile body condition in Seychelles warblers predicts adult condition and performance [46]. Juvenile and adult mass decrease in more competitive environments [39,47], and adult mass is positively correlated with food abundance [48].

## 2. Material and methods

### (a) Study population

Seychelles warblers are insectivorous passerines [49] endemic to the Seychelles archipelago. The population of approximately 320 birds in approximately 115 territories [34,50] on Cousin Island (29 ha, 04°20′ S, 55°40′ E) has been intensively monitored since 1997 [24,51], with virtually all breeding attempts followed each year during the major breeding season (June–September) and during most minor breeding seasons (January–March). Seychelles warblers are territorial and form socially monogamous pairs that defend the same territory year-round [24,52], but can be accompanied by one or more adult subordinates of either sex [52,53]. The dominant breeding pair is identified every season using clear courtship behaviour unique to dominant breeders, including contact calls and mate guarding [30,51]. Subordinates are assigned as helpers or non-helpers based on whether they incubate and/or provision offspring in the breeding territory [24,51]. Individuals can be reliably assumed dead when they are not observed for two consecutive seasons, as inter-island dispersal is virtually absent (<0.1% of individuals in their lifetime [31]) and resighting rates are very high (98% ± 1% s.e. for adults [32,33]).

### (b) Partnerships

Parental pair-bonds can last between one breeding season and a lifetime, with the longest recorded partnership being 15 years [26]. Partnerships can end (between or within breeding seasons) in different ways; most commonly through the death of one partner (widowhood) or both. Partnerships can end in divorce, whereby both partners are still alive but at least one partner lost the dominant breeding position in the focal territory. Partnerships were assessed throughout each breeding season and the partnership statuses assigned at the end of each breeding season. A partnership can have six different transitions from the end of one breeding season (major or minor) to the end of the next: (i) pair-bond fidelity (stay together), (ii) widowhood by female’s death, (iii) widowhood by male’s death, (iv) death of both partners, (v) divorce, and (vi) translocation. Divorces caused by one breeder ‘stepping down’ to a subordinate position for a single breeding season while still present in the territory were removed from our dataset (*n* = 22 out of 1362 pair-bond observations). Pair-tenure was defined as the duration from the start date of the first season the pair were pair-bonded to each other, until the end of the last breeding season when the focal offspring was reared.

### (c) Reproduction

During the major breeding season, 91% of pairs attempt to breed [54]. A maximum of one clutch is produced per breeding season and most clutches have a single egg, with only 8% of clutches having 2−3 eggs [26]. When nests contain multiple eggs, this usually indicates co-breeding subordinate females within the territory [51,55], which account for 11% of all maternities in the population [56]. Extra-pair fertilizations are common in the Seychelles warbler, with approximately 44% of all offspring being sired by a dominant male other than the socially pair-bonded male [55–57], although this is rarely a within-group extra-pair male (0.01% of fathers are within-group males [34]). Offspring remain dependent on parental care for approximately 3 months, long after fledging after approximately 18 days and survival during this period is dependent on the amount of provisioning that is received [27].

### (d) Individual sampling

Each breeding season, as many birds as possible are caught using mist nets or captured as nestlings in the nest, after which they are ringed with a unique BTO ring and three colour rings, and blood sampled (approx. 50 µl) from the brachial vein. Individuals caught for the first time are aged based on lay, hatch or fledge date and/or eye colour [49]. Their body mass is measured using an electronic scale (±0.1 g) and their structural size is assessed (tarsus length) using sliding callipers (±0.1 mm), resulting in 598 mass and size measures in 497 offspring. Haematocrit was assessed for 456 individuals (579 samples) by measuring the proportion of erythrocytes relative to the whole-blood volume using sliding callipers (±0.01 mm), on a microcapillary tube centrifuged within 3 h of collection for 8 min at 6000*g* [43]. A small subset of the blood sample (approx. 10 µl) is stored in absolute ethanol, and used for molecular sexing, parentage assignment and telomere length measurement. Genetic parents were assigned (*p* ≥ 0.8) using MasterBayes 2.5.2 [34,57]. Our data included all 1109 individuals that hatched between 1997 and 2018 and were assigned a pair-bonded mother. We used available telomere data for offspring that were sampled between 1997 and 2014. Samples that did not show signs of degradation were used for relative telomere length (RTL) assessment using quantitative polymerase chain reaction [34,36], and samples that did not meet quality criteria were removed [34,38]. RTL in the Seychelles warbler decreases extremely rapidly between 0 and 40 days old, after which it stabilizes [36]. Hence, we excluded offspring younger than 40 days, resulting in 661 RTL samples for 546 offspring. Within-plate repeatability was 0.74 (95% CI = 0.74−0.75) for GAPDH and 0.73 (95% CI = 0.71–0.74) for telomere Cq values, and between-plate repeatability was 0.68 (95% CI = 0.65–0.70) using 422 samples measured at least twice at different time points [36].

### (e) Statistical analyses

We performed all statistical analyses using *R* 4.3.0 [58]. Models were fitted with lme4 1.1.13 [59] or glmmTMB 1.1.17 [60]. For all models, we first z-transformed (mean centred and divided by 1 s.d.) all continuous predictors and checked for collinearity between fixed effects using variance inflation factor (all < 3) using performance 0.10.8 [61], checked for under- or overdispersion, and residual spatial or temporal autocorrelation using DHARMa 0.4.6 [62], finding none in the final models. While all first-order factors were kept in the model irrespective of their significance, all non-significant interactions were removed from the models sequentially, least significant first, to facilitate interpretation of the first-order effects. As we were interested in the effects of both pair-bond tenure and ending, we included both parental pair-bond tenure (in days) and ending categories (stay together: yes/no). In separate models, we separated pair-bond ending into more specific categories: (i) pair-bond fidelity (i.e. stay together), (ii) widowhood by female’s death, (iii) widowhood by male’s death, (iv) death of both partners, (v) divorce and (vi) translocation. When there were less than six observations of a pair-bond ending category for an analysis, it was removed from the corresponding model.

We first assessed the effect of parental pair-bond ending on condition measures of fledged offspring using linear mixed models (LMMs) with a Gaussian error distribution.

#### (i) Telomere length (relative telomere length) models

RTL was square-root transformed and z-transformed as a response variable to be consistent with previous protocols in this study population [34,36]. We fitted all offspring RTL measures and included log-transformed offspring sampling age since RTL changes loglinearly with age [36], offspring sex, parental pair-bond tenure, pair-bond ending category, paternal and maternal age since RTL increases with maternal age and decreases with paternal age [34], helper presence in the natal territory (present/absent) since alloparental care can alleviate reductions in RTL [63], whether the offspring was sired by an extra-pair father (yes/no) or offspring of a cobreeder (yes/no) to control for possible effects of extra-pair parentage, and technician identity (two levels, A/B) [21]. We tested for interactions between offspring age and sex and both parental pair-bond tenure and ending category, since the effects of parental pair-bonds on RTL may be age- and sex-dependent [34,36]. As random effects, we added offspring, mother and father identities, hatch season identity (i.e. season in which the offspring hatched) and qPCR plate [21].

#### (ii) Haematocrit models

In the models with haematocrit as a response variable, we included fixed effects: offspring sampling age (as linear and squared variables) since haematocrit changes quadratically with age in juveniles [43], parental pair-bond tenure, pair-bond ending category, offspring sex since juvenile haematocrit levels are sex-dependent [43], helper presence in the natal territory (present/absent) to control for alloparental effects, whether the offspring was sired by an extra-pair father (yes/no) or offspring of a cobreeder (yes/no) to control for possible effects of extra-pair parentage and time of day of sampling as this influences haematocrit [43]. We also tested interactions between age (linear and quadratic) and pair-bond tenure and pair-bond ending, respectively, as we expect an age-dependent effect of pair-bond tenure and pair-bond ending on haematocrit. As random effects, we included offspring, mother and father identity and hatch season.

#### (iii) Body condition models

As fixed effects in the models with body mass as a response variable, we included offspring structural size (tarsus length) as we are specifically interested in body condition, offspring sampling age since many juveniles were still receiving parental provisioning [27,28], offspring sex since male Seychelles warblers are *ca* 10% heavier than females [46], parental pair-bond tenure, pair-bond ending category, helper presence in the natal territory (present/absent) to control for alloparental effects and whether the offspring was sired by an extra-pair father (yes/no) or offspring of a cobreeder (yes/no) to control for extra-parentage effects. As random effects, we included offspring, mother and father identity and hatch season.

#### (iv) Lifespan and LRS models

In our models of long-term offspring fitness proxies, we included offspring that survived to independence (*n* = 610), i.e. being at least 3 months of age. We did this since most birds only get ringed after this age because nests and fledglings located high in the canopy are hard to reach [64]. The response variables were either lifespan or LRS. Since Seychelles warblers have sex-specific variation in lifespan and LRS and parental effects on offspring lifespan and LRS can be offspring sex specific [21], we expect the effect of parental pair-bonds on LRS and lifespan to differ between the sexes. Therefore, we used sex-specific generalized linear mixed models (GLMMs) with a negative binomial error (lifespan) and zero-inflated negative binomial (LRS) error distributions. We included two proxies of the natal social environment: helper presence since this is associated with LRS [21] and group size (number of adults within the territory; range: 2−7) to differentiate between the effects of additional individuals (subordinates) and alloparental care. Since only some subordinates help (20% of males and 42% of females), these variables are not highly correlated [29]. We added sibling presence to account for differences in lifespan and reproductive potential between siblings and singletons [47], and whether the offspring was sired by an extra-pair father (yes/no) or offspring of a cobreeder (yes/no) to account for extra-pair effects. We also included maternal age at conception as this is associated with lifespan and LRS [21]. As random effects, we added offspring, mother and father identity. Finally, we included hatch season as both a random effect to account for variation caused by hatch season identity and a fixed effect since offspring hatched more recently in our data lived relatively shorter lives, as offspring still alive at the last year of sampling were excluded from these analyses [65]. To calculate the hazards ratio for parental pair-bond tenure and ending on lifespan, we ran a Cox mixed-effects proportional hazards model using *coxme* 2.2.18.1 [66]. We used the same random and fixed effects as above but excluded hatch season, since individuals still alive at the latest sampling date (*n* = 114) or translocated to another island (*n* = 34) were right censored. The year of death was defined as the first year in which the individual was no longer seen. We confirmed that assumptions of proportional hazards were met using Schoenfeld’s residuals [67].

## 3. Results

Of the 1109 offspring hatched between 1997 and 2021, 872 (75%) had parents that remained pair-bonded to the end of the next season (after offspring became independent). In the remaining cases, the parental pair-bonds ended before the end of the next season as a result of maternal death 87 (8%), paternal death 81 (7%), both parents’ death 37 (3%), divorce 18 (2%) and translocation of one parent to another island 15 (1%) times.

### (a) Parental pair-bonds and juvenile-stage fitness proxies

**Figure 1.**
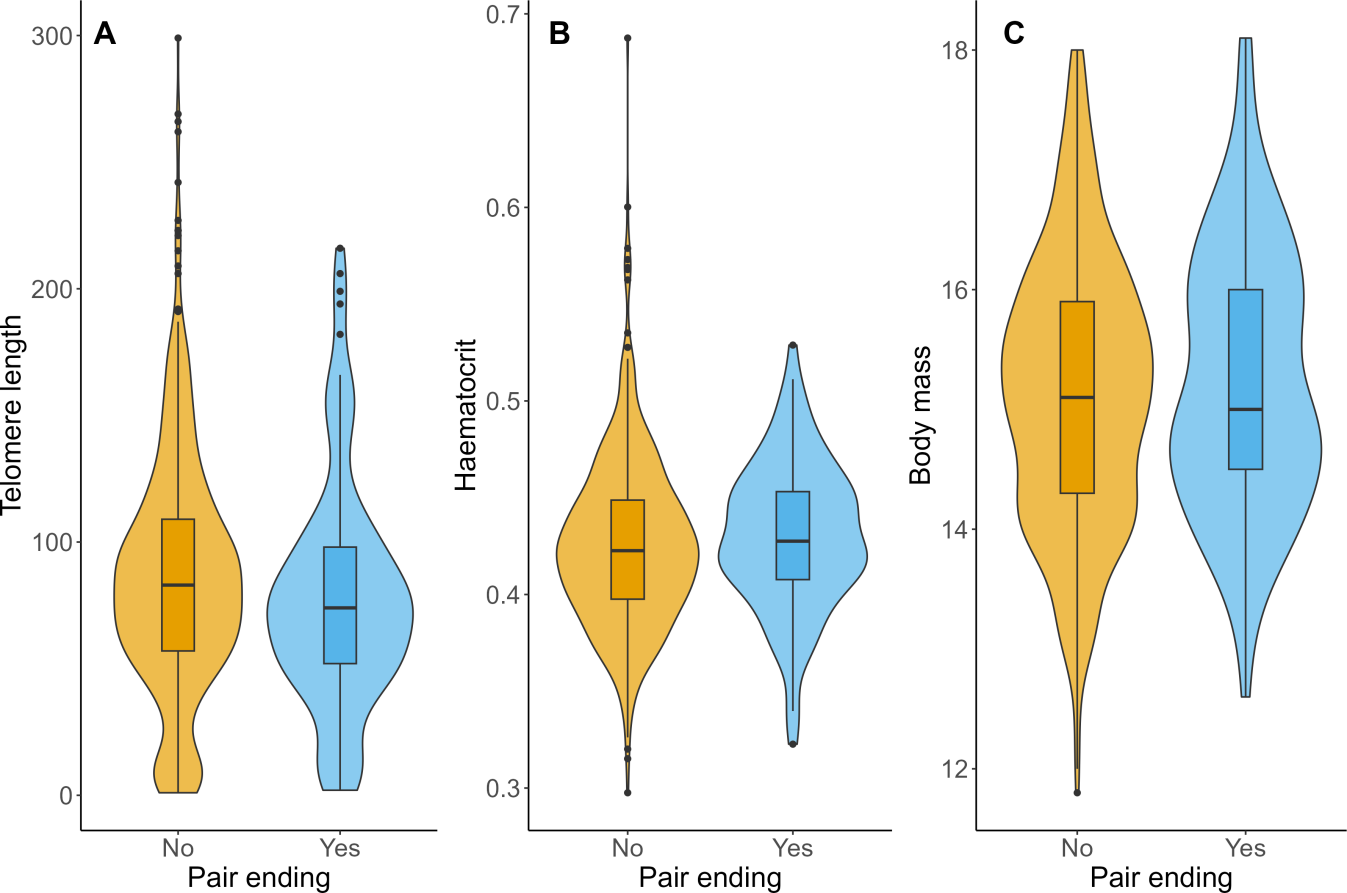
Violin plots of the juvenile-stage fitness proxies in relation to whether the parental pair-bond ended (yes/no), for (A) telomere length, (B) haematocrit and (C) body mass.

Early-life relative telomere length (RTL; *n* = 661), a biomarker of physiological condition, was significantly lower for offspring whose mothers died (table 1), although this was based on a small sample size (*n* = 33). We found no evidence that RTL was associated with parental pair-bond tenure (table 1; electronic supplementary material, table S1), nor pair-bond ending (yes/no; figure 1A; electronic supplementary material, table S1). We found no significant association between early-life offspring haematocrit (*n* = 579, electronic supplementary material, tables S2 and S3), nor body condition (*n* = 598, electronic supplementary material, tables S4, S5) with parental pair-bond tenure or parental pair-bond ending category (figure 1B,C). Although all three measures (RTL, haematocrit, body condition) were significantly associated with age, there was no interaction effect with age and parental pair-bond tenure or ending. Juvenile males had lower haematocrit (electronic supplementary material, tables S2 and S3) and better body condition than juvenile females (electronic supplementary material, tables S4 and S5).

**Table 1.** Linear mixed model results on the effect of parental pair-bond tenure and pair-bond ending on early-life relative telomere length in offspring in the seychelles warbler (*n* = 666). Significant fixed effects (*p* < 0.05) are in bold. Reference categories are pair-bond ending = yes (remain together), sex = female, helper presence = no, offspring sired by extra-pair male (EPP) = no, offspring of cobreeder = no, technician ID = A.

fixed effects		estimate	s.e.	*t*	*p*
intercept		0.918	0.316	2.903	0.004
pair-bond ending	yes	−0.124	0.104	−1.186	0.236
**offspring age**		**−0.410**	**0.144**	**−2.846**	**0.006**
maternal age		0.004	0.047	0.086	0.931
paternal age		0.003	0.047	0.057	0.955
sex	male	−0.021	0.104	−1.186	0.236
helper presence	yes	0.022	0.106	0.206	0.837
EPP	yes	0.006	0.079	0.078	0.938
cobreeder	yes	0.125	0.165	0.757	0.449
pair-bond tenure		0.009	0.041	0.219	0.827
technician ID	B	0.076	0.128	0.598	0.550

random effects	s.d.	*n*
offspring ID	0.212	550
mother ID	0.179	261
father ID	0.059	263
hatch season	0.246	30
plate ID	0.104	57
residual	0.917	

### (b) Parental pair-bonds and long-term offspring fitness proxies

Neither parental pair-bond tenure nor ending were associated with male (*n* = 288) and female (*n* = 322) offspring lifespan (Cox mixed-effects proportional hazards model: table 2; electronic supplementary material, table S6), even when excluding offspring that were still alive (*n*_male_ = 232, *n*_female_ = 251; GLMM: electronic supplementary material, table S7). LRS was also not predicted by parental pair-bond tenure and ending (*n*_male_ = 171, *n*_female_ = 250; table 3; electronic supplementary material, table S8). Only hatch season predicted offspring lifespan and LRS (table 3; electronic supplementary material, tables S7, S8), but this effect was likely driven by offspring that were still alive (and thus hatched in a more recent season) being removed from the dataset leading to only offspring from these recent seasons with shorter lifespans being included in the dataset.

**Table 2.** Cox mixed-effects proportional hazards model results including the hazards ratio (HR) on the effect of parental pair-bond tenure and ending on offspring lifespan in the Seychelles warbler in (A) females (*n* = 322) and (B) males (*n* = 288) using a binary variable for pair-bond ending (yes/no). Each pair-bond ending category includes the sample size in brackets. Reference categories are pair-bond ending = no (remain together), helper presence = no, offspring sired by extra-pair male (EPP) = no, offspring of cobreeder = no, sibling presence = no.

		(A) females				(B) males			
fixed effects		HR	s.e.	*z*	*p*	HR	*s.e.*	*z*	*p*
pair-bond ending	yes	1.045	0.183	0.24	0.810	0.887	0.223	−0.53	0.590
maternal age		0.989	0.072	−0.15	0.880	1.069	0.092	0.73	0.470
helper presence	yes	1.329	0.160	1.77	0.076	0.880	0.226	−0.57	0.570
EPP	yes	1.021	0.130	0.16	0.870	1.026	0.165	0.15	0.880
cobreeder	yes	1.078	0.138	−0.47	0.590	1.293	0.262	0.98	0.330
sibling presence	yes	1.078	0.138	0.54	0.590	0.772	0.198	−1.31	0.190
group size		0.977	0.075	−0.04	0.960	1.029	0.105	0.27	0.780
pair-bond tenure		1.019	0.073	0.26	0.800	0.985	0.092	−0.16	0.870

random effects	s.d.	*n*	s.d.	*n*
mother ID	0.020	202	0.212	155
father ID	0.020	203	0.040	163
hatch season	0.240	36	0.284	31

**Table 3.** Zero-inflated generalized linear mixed model results of the effect of parental pair-bond tenure and ending on offspring lifetime reproductive success in the Seychelles warbler in (A) females (*n* = 250) and (B) males (*n* = 171) using a binary variable for pair-bond ending (yes/no) excluding offspring that are still alive. Significant fixed effects (*p* < 0.05) are in bold. Reference categories are pair-bond ending = no (remain together), helper presence = no, offspring sired by extra-pair male (EPP) = no, offspring of cobreeder = no, sibling presence = no.

		(A) females				(B) males			
fixed effects		estimate	s.e.	*z*	*p*	estimate	s.e.	*z*	*p*
intercept		1.233	0.197	6.265	<0.0001	0.548	0.288	1.903	0.057
zero-inflated intercept		0.050	0.190	0.263	0.793	−1.515	0.916	−1.653	0.098
pair-bond ending	yes	−0.307	0.256	−1.198	0.231	−0.033	0.332	−0.101	0.920
mum age		−0.038	0.096	−0.399	0.690	0.080	0.147	0.545	0.586
helper presence	yes	−0.425	0.234	−1.819	0.069	0.303	0.327	0.926	0.354
EPP	yes	−0.280	0.179	−1.570	0.117	−0.062	0.265	−0.235	0.814
cobreeder	yes	0.157	0.264	0.595	0.552	0.587	0.355	1.658	0.098
sibling presence	yes	−0.026	0.176	−0.148	0.882	−0.205	0.312	−0.658	0.511
group size		−0.085	0.110	−0.777	0.437	−0.057	0.157	−0.365	0.715
pair-bond tenure		0.160	0.092	1.730	0.084	0.101	0.138	0.737	0.461
hatch season		**−0.581**	**0.121**	**−4.809**	**<0.001**	**−0.677**	**0.177**	**−3.832**	**<0.001**

random effects	s.d.	*n*	s.d.	*n*
mother ID	0.00004	161	0.00005	129
father ID	<0.00001	165	0.00007	137
hatch season	<0.00001	31	0.4428	31
breeding group	0.0001	236	0.00004	163

## 4. Discussion

This study adds to the growing body of literature on short- and long-term parental effects on offspring fitness [17,68], and, to our knowledge, is the first to test for long-term effects of parental pair-bonds in species other than humans [22,23]. We found no convincing evidence of parental pair-bonds being associated with short-and long-term fitness components in the Seychelles warbler. Telomere length in the first year of life was lower for offspring whose mother died, although this analysis had low statistical power. Besides this finding, telomere length, haematocrit and body condition in the first year of life were not associated with parental pair-bond tenure or ending, nor were lifespan and LRS.

The effect of widowhood by the death of the female on offspring telomere length could be driven by increased stress experienced by the offspring early in life, meaning they face a reduction in telomere length. Maternal effects on offspring telomere length have been found before in Seychelles warblers, with females surviving to older ages producing offspring with longer telomeres [34]. However, our finding is based on a small sample size of female deaths (*n* = 33), meaning we have limited power to support this finding. Additionally, this effect did not exist in any of the other measures of offspring condition early in life (haematocrit and body condition), nor did it result in detrimental effects later in life for the offspring (lifespan, LRS). Thus, we do not claim that mother death results in changes in offspring fitness.

We found no evidence of a relationship between parental pair-bond ending on the other short- and long-term offspring fitness condition measures. This suggests that the ending of a parental pair-bond is not a major component of the socio-environmental stressors experienced by offspring. Seychelles warblers that divorce or are widowed usually re-pair by the next breeding season [26]. As the population is highly saturated and there is strong competition for breeding vacancies [50,69], the breeding position may be filled up very quickly, sometimes within hours [24,69]. These new replacement breeders may alleviate potential stress experienced by the original breeder providing care to the dependent offspring. Additionally, the presence of helpers is positively associated with the lifespan of dominant female breeders [29] and alleviates the costs of parental care for ageing female Seychelles warblers [27,70], suggesting that helpers may alleviate the costs of loss of parental care from the ending of the parental partnership (although they do not take over the breeding position themselves [71]). However, we did not find any effect of helper presence on offspring fitness components. To what extent helpers may adjust their levels of parental care as a response to one of the dominant breeders disappearing (through divorce or death) remains to be investigated. An alternative explanation for this lack of a consistent detectable effect on telomere length, body condition, and haematocrit is that these measures are not sensitive enough to capture the stress juveniles experience due to having parents with little pair-breeding experience or following parental pair-disruption. However, telomere length, body condition [46] and haematocrit [43] all correlate with other early-life environmental factors, and, subsequently with survival, in the Seychelles warbler.

Parental pair-bond duration was not associated with any offspring fitness components in the Seychelles warbler, suggesting that parental pair-breeding experience does not strongly affect offspring on the short- and long term. When controlling for age, partners who have been together for a long time either do not yield higher quality offspring, compared with those with newer partners, or effects on offspring in very early-life condition do not translate into any long-term effect on fitness components in the present study. However, early-life sociological and environmental conditions have been linked to short- and long-term fitness components in the Seychelles warbler using the same metrics in the present study [21,36,46,70]. This suggests that although fitness components used in this study are affected by the early-life environment, they are not affected by parental pair-bond tenure specifically. Additionally, disruption of the pair-bond when offspring are still dependent on their parents and/or the parental territory did not negatively affect the long-term performance of these offspring in this study. Parental pair-bond tenure may yield other benefits rather than increasing offspring quality, such as higher annual survival of the parents [9,72]. In the Seychelles warbler, pair-bond tenure and pair-bond disruption through widowhood and divorce are not associated with individual reproductive success, but females that are divorced and lose their breeding position have lowered survival compared with females that stayed in their partnership [26]. Altogether, these results suggest pair-bonded Seychelles warblers do not yield significant synergistic reproductive benefits by staying and breeding together in terms of offspring quantity or quality. In other systems where individuals form long-term socially monogamous partnerships, the parental pair-bond may have larger effects on offspring fitness. For instance, if there is very little or no extra-pair parentage meaning both parents are always benefitting from raising offspring and may invest more in the offspring as a result, exclusive parental care by the partners (i.e. no cooperative breeding), and a long period of offspring dependence meaning offspring are highly dependent on extended care of both parents, there may be a stronger effect of the parental pair bond on offspring fitness. This provides an interesting avenue to study the effects of socially monogamous partnerships on offspring fitness in species that fit these criteria.

Finally, it is possible that parental pair-bond tenure and pair-bond ending do affect offspring phenotype and fitness in Seychelles warblers, but only in very early-life during the egg and hatchling stage. In this case, the effect will not be detected by our study since we are often unable to assess eggs or nestlings at very early stages in this system due to the inaccessibility of many nests. It could be that the adversity of pair-bond disruption and/or short pair-bond tenure means that parents experiencing these are more likely to lose their offspring very early on (as eggs or hatchlings), or do not attempt to breed in that season. Thus, these parents will not be captured in our study (i.e. selective disappearance). Investment in egg composition is commonly influenced by maternal effects [73,74], often mediated by maternal deposition of hormones in the egg [75] and maybe one route by which pair-bonds may impact egg or nestling quality. Furthermore, chicks are entirely dependent on parental care during the nest stage in altricial birds, which may also be negatively affected by pair-bond factors leading to selective disappearance. Post-natal parental care is argued to be the most important maternal effect during this period [76], so much so that it can mask other maternal effects such as investment in the egg [77]. However, divorcing and widowhood in the Seychelles warbler are not associated with reproductive output at the egg-laying or fledgling stage [26], thus, any strong parental effect on offspring survival prior to the point at which they are included in this study, appears unlikely.

## 5. Conclusion

This study highlights the surprising lack of intergenerational effects of social breeding partnerships on fitness in the Seychelles warbler. Although social monogamy is a common mating system, especially in avian taxa [1], the implications of staying with the same partner for extended periods on offspring fitness are understudied. This study sets the groundwork for quantifying long-term parental effects of mate switching and pair-bond tenure in socially monogamous breeders. We hope this study stimulates future research to test if the lack of influence of parental pair-bonds on offspring fitness components that we found is ubiquitous across taxa. A focus on the potential mechanisms through which long-term partnerships in socially monogamous species may have intergenerational effects may provide important insights. Additionally, experimental studies will allow for elucidating the causal link between parental pair-bond and offspring fitness proxies.

## Data Availability

Data and code are available on University of Groningen Dataverse [78]. Supplementary material is available online [79].
